# Content Development for a Virtual Social Engagement Intervention

**DOI:** 10.1093/geroni/igab046.1647

**Published:** 2021-12-17

**Authors:** Shraddha Shende, Allura Lothary, Justine King, Sarah Jones, Raksha Mudar, Dillon Myers, Wendy Rogers

**Affiliations:** 1 University of Illinois Urbana-Champaign, Champaign, Illinois, United States; 2 University of Illinois Urbana-Champaign, Urbana, Illinois, United States; 3 iN 2.L, Greenwood Village, Colorado, United States; 4 University of Illinois-Urbana Champaign, Champaign, Illinois, United States; 5 OneClick.chat, Philadelphia, Pennsylvania, United States

## Abstract

Video technology has the potential to provide older adults with socially and cognitively engaging activities for in-home participation. We are exploring use of OneClick.chat, a video technology platform, to present older adults with and without mild cognitive impairment opportunities for engagement. In collaboration with iN2L we have developed events that will facilitate conversations that do not rely on episodic memory, cover a range of topics, and represent different cultures and interests. We selected event topics that were positive, socially and cognitively engaging, and included a range of pictures based on our previous research. Events were carefully controlled for length of presentation, picture type, and readability. Discussion questions related to the events were designed to stimulate engaging conversations through open-ended questions and to not burden memory recall or enforce stereotypes. Our work highlights potential future avenues for researchers and home and community-based organizations to use technology to promote social engagement.

